# Unveiling Associations of COVID-19 Vaccine Acceptance, Hesitancy, and Resistance: A Cross-Sectional Community-Based Adult Survey

**DOI:** 10.3390/ijerph182312348

**Published:** 2021-11-24

**Authors:** Carmina Castellano-Tejedor, María Torres-Serrano, Andrés Cencerrado

**Affiliations:** 1 Psynaptic, Psicología y Servicios Científicos y Tecnológicos S.L.P, 08192 Barcelona, Spain; acence@gmail.com; 2 GIES Research Group, Basic Psychology Department, Autonomous University of Barcelona, 08192 Barcelona, Spain; 3 Research Group on Aging, Frailty and Care Transitions in Barcelona, Parc Sanitari Pere Virgili & Vall d’Hebron Research Institute (VHIR), 08023 Barcelona, Spain; 4 Faculty of Psychology, Autonomous University of Barcelona, 08192 Barcelona, Spain; torresserranomaria23@gmail.com

**Keywords:** COVID-19, pandemic, vaccination, resistance, hesitation, online survey, cross-sectional survey

## Abstract

COVID-19 vaccines are essential to limit and eliminate the infectious disease. This research aims to identify strong vaccination resistance profiles and/or hesitation considering health, psychosocial, and COVID-related variables. A cross-sectional online survey (*N* = 300) was conducted in the context of strict COVID-related gathering and mobility restrictions (January–March 2021). Data collected were vaccine acceptance, hesitancy and resistance rates, general psychosocial status, and preventive practices and beliefs regarding COVID-19 and its vaccination, among other factors. Logistic regression was applied to a real-world data set and a significant model (χ^2^ (7, *N* = 278) = 124.548, *p* < 0.001) explaining 51.3% (R^2^ Nagelkerke) of attitudes towards vaccination was obtained, including the following predictors for acceptance: to have greater confidence in the COVID vaccine security (OR = 0.599) and effectiveness (OR = 0.683), older age (OR = 0.952), to be a healthcare professional (OR = 0.363), to have vulnerable individuals in charge (OR = 0.330), and sustain the belief that the vaccine will end the pandemic situation (OR = 0.346) or not being sure but give some credence to that belief (OR = 0.414). Findings could help understand the rate and determinants of COVID-19 vaccine resistance/hesitancy among a Spanish population sample and facilitate multifaceted interventions to enhance vaccine acceptance.

## 1. Introduction

Severe acute respiratory syndrome coronavirus 2 (SARS-CoV-2) has caused the COVID-19 pandemic, as established by 11 March 2020. As of June 2021, the virus had spread to more than 200 countries, infected over 176 million people, and resulted in over 3 million deaths worldwide. Over the next two years, the global socio-economic costs of this pandemic are expected to be enormous. Many families and societies would receive a substantial hit, leading to cumulative losses and increasing prevalence of psychopathology [1], especially among those at higher risk [2,3,4]. In addition, several studies on the mental health impact of COVID-19 have found a high risk of mental disorders among young adults (<40 years of age), young students, women, those with high levels of exposure to COVID-19-related media communications [5], as well as healthcare professionals working in the frontline and those having experienced the death of a close person by COVID-19 [6,7].

In the beginning, and in the absence of an effective therapy or vaccine, governments worldwide enacted physical distancing and quarantine measures to slow the spread of the virus, protect the most vulnerable in society, and manage health care service demand and provision [8]. The first human clinical trial of a COVID-19 vaccine commenced in March 2020 in the United States [9], and several other human trials began soon after. As of September 2020, 8 vaccines had advanced to phase 3 clinical trials, and two others had been approved for early or limited use. Currently, just in Western Europe, four different vaccines are available with different regulations according to each country and region (Pfizer-BioNTech, Moderna, AstraZeneca, and Janssen), and all of them outweigh the known and potential risks. Worldwide, more vaccines are available and continue to be developing and testing. Despite promising results, there is general agreement that clinical trials would provide long-term follow-up data to support the eventual full licensure of all these vaccines. The best randomized clinical trial design is a double-blind crossover trial in which all participants would receive the other intervention that they were not given at the start of the trial (i.e., the vaccine or the placebo). However, reality makes it impossible to follow this design for all vaccines at this stage and different designs have been carried out instead.

In 2019, the World Health Organization (WHO) already identified ten threats to global health. Among these threats, vaccine hesitancy, the risk of a worldwide influenza pandemic, and the risk of emergence of high-threat pathogens such as Middle East respiratory syndrome (MERS) and/or severe acute respiratory syndrome (SARS) were identified [9]. One year later, this WHO statement, via COVID-19, emerged, and the race to get a safe and effective vaccine quickly started. The minimum standard time to develop a vaccine is estimated to be between 1–1.5 years, as different steps are necessary during the clinical development. Among these steps, recruitment of volunteers in a vaccine clinical trial is a real challenge, and sometimes, some trials must be stopped due to difficulties in recruitment [10,11]. After its clinical development, a vaccine also faces the challenge of acceptance by the general population.

In April 2020, an online survey among representative samples of the population (in terms of region, gender, age group, and education) was carried out in seven European countries (*N* = 7.662; Denmark, France, Germany, Italy, Portugal, the Netherlands, and the United Kingdom). This research found that up to 18.9% of respondents were unsure about taking a vaccine for COVID-19, and, most importantly, 7.2% stated that they did not want to get vaccinated [12]. To date, some research has explored different psychological factors concerning vaccine hesitancy and resistance (COVID-19 or not COVID-19 vaccine-related), for instance: personality traits such as neuroticism and conscientiousness [13,14], altruism [15], locus of control [16], and cognitive reflection [17]. In addition, previous research has put forward some relationships associated with conspiratorial, religious, and paranoid beliefs [18,19]. Similarly, mistrust of authoritative members of society such as government officials, scientists and health care professionals have been linked to negative attitudes towards vaccinations [20,21,22].

Taken all together, the existing literature indicates that there are likely to be several individual psychological dispositions that traverse personality, emotional and cognitive styles, beliefs, and socio-political attitudes that distinguish those who are more prone to take positions ranging from being unsure about taking a vaccine or being absolutely against taking it, from those who are more prone to take positions ranging from passive acceptance to active demand. Despite all this evidence, research specifically focused on COVID-19 vaccines resistance or hesitancy is still scarce [23]. However, emerging research findings stress that a substantial proportion of European adults are hesitant about/resistant to a vaccine for COVID-19 [12,23]. As further emphasized many years before by the WHO Strategic Advisory Group of Experts (SAGE) on Immunisation [24,25], substantial work is required to begin to understand and address this problem and also to aid future public health messaging [26,27].

To explore psychological characteristics associated with COVID-19 vaccine hesitancy and resistance, this study aimed (1) to determine what proportions of the general adult populations of Spain were accepting of, hesitant about, or resistant to a vaccine for COVID-19 in the context of a broad population lockdown; and (2a) to profile individuals who are hesitant about/resistant to a possible vaccine for COVID-19 by identifying the key sociodemographic, psychosocial, and health-related factors that distinguish these individuals from those who are accepting of a COVID-19 vaccine and (2b) identify the most salient psychological characteristics of both groups.

All this knowledge will offer a more comprehensive understanding of how public health officials can effectively tailor health behavior messaging to align with the psychological profiles and dispositions of vaccine-hesitant/resistant individuals, achieving public health campaigns and their objectives in a more efficient manner.

## 2. Materials and Methods

### 2.1. Design

This study follows an exploratory cross-sectional population survey design.

### 2.2. Study Subjects and Sampling

A non-probabilistic snowball sampling of community-based adults was recruited for the study. Inclusion criteria required being ≥18 years old. Exclusion criteria were not understanding Spanish well enough to complete the questionnaire. These criteria were stated in the informed consent presented before the survey. In addition, questionnaires submitted below the cut-off of 4 min were excluded from the survey. Nonprobability sampling (convenience sampling) was adopted for the study as it requires much less time and effort and supports cost minimization.

A total of 300 adults from different Spanish provinces (Barcelona 65.33%, *n* = 196) filled the survey (21 cases were excluded for not meeting inclusion/exclusion criteria). The majority of the sample lived in the region of Catalonia, with just a few cases being abroad (3%, *n* = 9) at the moment of the study. Sociodemographic characteristics are comprehensively presented in Table 1. Most respondents were women (75.7%, *n* = 227) aged 40.11 ± 12.34 (range 18–74), married or cohabiting with a partner (64%, *n* = 192), and in charge of vulnerable individuals (21.7%, *n* = 65).

### 2.3. Procedure and Data Collection

Between 24 January and 21 March 2021 in a context of strict general social gathering and mobility restrictions, a brief snowball survey entitled: “Personal attitudes towards the COVID-19 pandemic” was distributed via mailing lists and social media (WhatsApp, Facebook, and Twitter). Participants were encouraged to distribute the survey with their contacts and relatives. We selected an online survey as the lockdown and enrollment of participants precluded in-person surveys and random selection.

Based on our previous research [3] and from a combination of the Health Belief Model (HBM) and Protection Motivation Theory (PMT) [28,29], a brief multifactorial 48-item questionnaire using the Kwik surveys platform was developed to examine potential factors related to attitudes towards COVID-19 vaccines. The survey was available only in Spanish, as we aimed to reach as many respondents as possible in our country and/or Spanish citizens living abroad but depending on the Spanish healthcare system. The survey contained several open-answer boxes to specify some responses and one final open-answer question asking respondents to offer more details concerning the topic assessed. The survey also included a review step and a completeness check before being able to submit it. We made the length of the survey short enough not to take more than 10 minutes to ensure a high completion rate. The Checklist for Reporting Results of Internet E-Surveys (CHERRIES) was employed (see Supplementary Material) [30].

Potential participants, pre-accessing the survey, were informed about the research objectives and the usefulness of the results. Main ethical and privacy details were outlined too, and permission to use data for scientific purposes was requested before entering the survey. Participants could withdraw at any time before submitting their final responses.

### 2.4. Variables and Assessment Measures

The study outcomes include factors associated with COVID-19 vaccine acceptance, resistance and hesitancy, and knowledge, attitude, and practices related to COVID-19 and vaccination. Data presented in this manuscript are part of a bigger research project exploring public perceptions, mass media information/misinformation, and personal attitudes towards the COVID-19 pandemic.

#### 2.4.1. Primary Outcome Variable

Item 15 assessed personal predisposition and readiness of being vaccinated for COVID (reading as follows: “Are you willing to be vaccinated for COVID-19?”) Optional answers were: *Yes; Probably yes; I haven’t decided yet; Probably not; No, just if I’m obliged; Depending on the vaccine; I have been already vaccinated; Do not know/Do not answer*. For the purposes of this research, and to define the three studied profiles, the sample was classified according to the following response options: (1) Acceptance (*Yes*), (2) Hesitant (*Probably yes; I haven’t decided yet; Probably not; Depending on the vaccine*), (3) Resistant (*No, just if I’m obliged*). Respondents stating *Do not know/Do not answer*, and *I have been already vaccinated* were not included in these comparisons.

#### 2.4.2. Other Variables

##### Socio-Demographics, Medical and Psychographic Characteristics

Participants’ socio-demographic data includes gender, age, residence area, nationality, cohabitation, employment status, and educational level. Medical and psychographic characteristics collected included: suffering from a chronic disease, psychological symptoms, receiving psychological support at the moment of the study (due or not to COVID), and if they have experienced the loss of a loved one due to COVID-19.

##### COVID-19-Related Data

Participants indicated whether they have been diagnosed with COVID-19, type of diagnostic test, need for quarantine and/or hospital admission (including ICU). Participants were also asked about the degree (VAS) to which they considered vaccines safe and effective, and the perception of it as the possible solution to end the pandemic caused by the COVID-19 (*yes* vs. *no* item).

##### COVID-19 Preventive Measures/Behaviors

Individual and voluntary preventative measures/behaviors were screened collecting the use of FPP2 masks, other masks, use of anti-COVID plastic face shields, transparent shields or other physical barriers on the desk/counter, hand hygiene with hydroalcoholic gel, surface hygiene with hydroalcoholic gel, wearing gloves, social distancing (1.5–2 m), restriction of departures at certain hours (curfew), limiting distance from home, limiting social gatherings to small groups, decreasing the frequency of attending to closed places, decreasing the frequency of attending crowded places for leisure, and other actions. Additionally, difficulties carrying out the different preventive measures/behaviors were also surveyed. Finally, compliance with preventive measures was assessed through a self-reported 5-point VAS where 1 meant *lousy* and 5 *excellent*.

##### Fear of COVID-19 Scale (FCV-19S)

The FCV-19S is a seven-item scale assessing the fear of COVID-19 using seven items rated on a 5-point Likert scale ranging from 1 *strongly disagree* to 5 *strongly agree*, with total scores ranging from 7 to 35 [31]. The present study showed the reliability of α = 0.854.

##### Generalized Anxiety Disorder-7 (GAD-7)

The GAD-7 is a seven-item self-report scale used in medical and community settings to screen the severity of generalized anxiety [32]. It exhibits excellent and robust psychometric properties in terms of validity and internal consistency (Cronbach’s alpha between 0.89 and 0.92) [33,34]. Using the GAD-7 scale, respondents were asked to answer how often they have been bothered by various anxiety symptoms over the last two weeks on a 4-point Likert scale ranging from 0 *not at all* to 3 *nearly every day*. The scale accordingly produced a total score ranging from 0 to 21. The present study showed the reliability of α = 0.899.

### 2.5. Ethics

No specific ethical approval was obtained since the authors’ research institutions do not oblige to for conducting surveys, as long as all ethical requirements are met. In this sense, the research ethics procedures of this study complied with European and national legislation (e.g., the Charter of Fundamental Rights of the EU, Directive 95/46/EC of the European Parliament and of the Council of 24 October 1995 on the protection of individuals concerning the processing of personal data and on the free movement of such data) and data were treated with confidentiality, equality, and justice, respecting the Helsinki principles. Researchers have also considered the American Psychological Association (APA) ethical principles of research conduct. Codification procedures were employed to ensure the privacy and confidentiality of information. All participants were informed about study purposes, and direct informed consent was requested from all respondents before starting the survey and sending their responses. No economic incentives were offered for taking part in the survey. Participants who required feedback from the survey were invited to write down their email addresses and receive information or specific helpful suggestions.

### 2.6. Statistical Analysis

Descriptive statistics were calculated for all outcome variables using measures of central tendency (mean, standard deviation, the range for continuous variables, frequencies, and total percentages for categorical variables). The Kolmogorov–Smirnov test was used for bivariate analysis to determine whether parametric or non-parametric tests were indicated. Bivariate comparisons were performed through either Student’s *t*-test or ANOVA for variables with more than two categories or levels, the Mann–Whitney U test for continuous variables, or the Chi-square test (and Fisher’s exact test when *n* < 5) for dichotomous variables. The correlation of variables was compared using Pearson correlation. Subsequently, logistic regression analyses (forward stepwise method) were performed to identify the key factors associated with vaccine acceptance and hesitancy. Considering the small sample size within the resistant group (*n* = 20), only descriptives were provided to characterize this subsample of individuals, not pursuing a logistic regression model to avoid statistical overestimation errors. The model was validated by (a) significant test of the overall model, (b) tests of regression coefficients, (c) goodness-of-fit measures, and (d) validation of predicted probabilities. All predictors (adjusted odds ratios; Exp (B)) were adjusted for all other covariates in the model. The level of statistical significance was 5% (*p* ≤ 0.05). In all cases, appropriate post hoc analyses were performed, and 95% confidence intervals were reported. All statistical analyses were performed using the SPSSv_25_ (IBM Corp., Armonk, NY, USA). Sample size calculation was based on a margin of error and confidence level rather than prevalence or expected effect sizes. With a 5% margin of error and a confidence level of 95%, a minimum sample size of 250 was estimated to be sufficient to reveal differences in an average response to each category concerning attitudes towards vaccination (the main outcome variable of the study).

## 3. Results

### 3.1. Objective 1: Prevalence of Vaccine Hesitancy, Resistance and Acceptance in Spain

**Figure 1:**
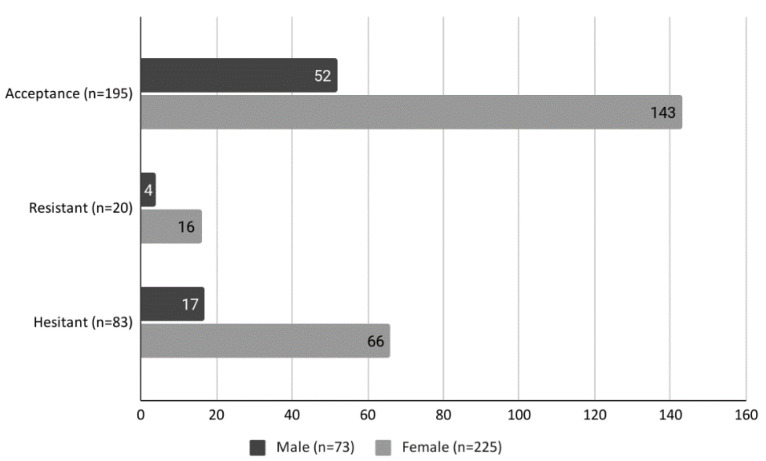
Prevalence of vaccine acceptance, hesitancy, and resistance according to gender.

Overall, 65.44% of respondents were willing to accept a COVID-19 vaccine (*n* = 195; 95% CI 59.83–70.64), 27.85% were hesitant (*n* = 83; 95% CI 23.04–33.24), and 6.71% were resistant to such a vaccine (*n* = 20; 95% CI 4.36–10.19). Figure 1 displays the proportions in these three groups according to gender where no statistically significant differences were observed.

Statistically significant differences were observed regarding age, with the elderly (>60 years old) significantly being more prone to accept vaccination (χ^2^ (4, *N* = 300 = 12.047, *p* = 0.017; 84.6%) compared to those aged between 36–59 (69.6%) and between 18–35 (55.3%). Similarly, healthcare professionals were also more explicit about the acceptance of the vaccine (χ^2^ (2, *N* = 300) = 12.868, *p* = 0.002; 76.7% vs. 56.8%).

### 3.2. Objective 2: Profile of Individuals Who Are Hesitant About/Resistant to a Possible Vaccine for COVID-19 by Key Socio-Demographic, Psycho-Social, and Health-Related Factors That Distinguish These Individuals from Those Who Are Accepting

Clinical and COVID-19-related data of the studied sample are comprehensively presented in Table 2.

There were no cases requiring hospital or ICU admission due to COVID.

Acceptance of the vaccine was related to having suffered the loss of a loved one due to COVID-19 (χ^2^ (2, *N* = 289)=15.621, *p* < 0.001; 78% of accepting individuals grieving for this situation in front of 14% of resistant and 8% of hesitant individuals), the belief that the effectiveness of the vaccine will resemble that of other already existing vaccines (χ^2^ (4, *N* = 300) = 40.047, *p* < 0.001; 76.9% vs. 44.4% that do not believe so) and, in addition, the belief that the vaccine will put an end to the current pandemic situation (χ^2^ (4, *N* = 300) = 40.228, *p* < 0.001; 81.8% vs. 41.9% that do not believe this). In addition, significantly higher scores were observed concerning the degree of confidence in the vaccine’s general security (*F*_(2)_ = 106.595, *p* < 0.001) among those more prone to accept being vaccinated (M ± SD = 7.76 ± 1.58; 95% CI 7.54–7.98) compared to those resistant (M ± SD = 5.42 ± 2.02; 95% CI 4.98–5.86) and hesitant (M ± SD = 2.68 ± 2.56; 95% CI 1.45–3.85). Similarly to what happens for self-perceived effectiveness of the vaccine (*F*_(2)_ = 88.235, *p* < 0.001), again with people accepting being vaccinated displaying higher scores (M ± SD = 7.67 ± 1.53; 95% CI 7.45–7.88) compared to those hesitant (M ± SD = 5.52 ± 2.01; 95% CI 5.08–5.96) and resistant (M ± SD = 3.25 ± 2.38; 95% CI 2.14–4.36).

Overall, FCV-19S scores were medium (M ± SD = 16.67 ± 5.44, range 7–34) with 42.7% of the sample (*n* = 128) scoring above 17.6 points (in a range 7–35) indicating moderate-to-high fear of COVID-19. People suffering from chronic diseases were significantly (*F*_(2)_ = 3.104, *p* = 0.046) more scared about COVID (M ± SD = 18.31 ± 5.12, 95% CI 16.92–19.70) than those who do not have any chronic condition (M ± SD = 16.32 ± 5.49; 95% CI 15,62–17,01), similar to what happens with those in charge of vulnerable individuals (*F*_(1)_ = 5.475, *p* = 0.020) (M ± SD = 18.06 ± 5.87; 95% CI 16.61–19.52) compared to those without such responsibility (M ± SD = 16.29 ± 5.27; 95% CI 15.61–16.97). Despite no significant differences were observed in expressed COVID-related fear among different profiles concerning attitudes towards vaccination, a tendency was observed (*p* = 0.051) among those more prone to accept getting vaccinated, displaying lower scores on the FCV-19S (M ± SD = 16.73 ± 5.43, 95% CI 15.96–17.50) compared to hesitant individuals (M ± SD = 17 ± 5.17, 95% CI 15.87–18.13), but revealing higher scores than resistant ones (M ± SD = 13.80 ± 5.41, 95% CI 11.27–16.33).

Generalized anxiety (GAD-7) scores were medium to low (M ± SD = 6.19 ± 4.88, range 0–21) with 82.3% of the sample (*n* = 247) referring mild anxiety (scores below 10), 11.7% (*n* = 35) moderate (scores between 11–15), and 6% (*n* = 18) severe anxiety. There were statistical significant differences between genders (*F*_(1)_ = 5.838, *p* = 0.016), with females revealing higher generalized anxiety (M ± SD = 6.57 ± 4.88; 95% CI 5.93–7.21) compared to males (M ± SD = 5 ± 4.71; 95% CI 3.90–6.10). In addition, those with vulnerable individuals at charge were significantly (*F*_(1)_ = 4.533, *p* = 0.034) more anxious (M ± SD = 7.32 ± 5.34; 95% CI 6–8.65) than those who do not have such responsibility (M ± SD = 5.88 ± 4.70; 95% CI 5.27–6.48).

Individual voluntary preventative measures/behaviors carried out during the strict lockdown, and self-perceived correctness performing them are presented in Table 3.

Self-perceived correctness performing preventative measures (VAS 0–5) was significantly higher (*F*_(2)_ = 3.163, *p* = 0.044) among resistant individuals (M ± SD = 4.20 ± 0.52; 95% CI 3.96–4.44) compared to those accepting (M ± SD = 4.16 ± 0.62; 95% CI 4.08–4.25) or hesitating (M ± SD = 3.96 ± 0.67; 95% CI 3.82–4.11) being vaccinated.

There were no statistically significant differences among the three profiles in terms of attitudes/behaviors to prevent COVID except for wearing gloves (χ^2^ (2, *N* = 300) = 10.077, *p* = 0.006) with a higher percentage of people accepting being vaccinated (82.9%) carrying out this behavior compared to 9.8% of resistant and 7.3% of hesitant individuals.

It was also observed that during the period of regulations/restrictions, males limited less social gatherings to reduced groups (χ^2^ (1, *N* = 300) = 4.025, *p* = 0.045; 17.8% men vs. 9.3% women not limiting such gatherings), avoided less attending crowded places for leisure (χ^2^ (1, *N* = 300) = 5.417, *p* = 0.020; 20.5% men vs. 10.1% women), and made fewer restrictions concerning departures at certain hours (χ^2^ (1, *N* = 300) = 4.159, *p* = 0.041; 43.8% vs. 30.8%) compared to females.

Similarly, individuals among 18–35 years old preferred to restrict less their outings (χ^2^ (2, *N* = 300) = 6.991, *p* = 0.030; 46.1% vs. 50% among 36–59 years old and 3.9% >60 years old, respectively) and social distancing (χ^2^ (2, *N* = 300) = 14.083, *p* = 0.001; 58.1% vs. 38.7% among 36–59 years old and 3.2% >60 years old, respectively) compared to other age groups.

**Figure 2:**
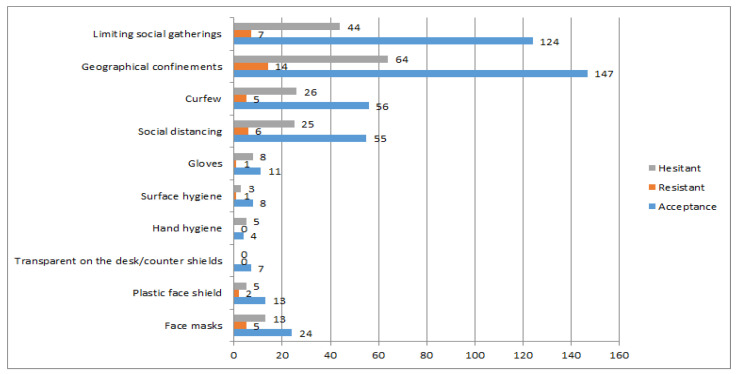
Most referred preventive measures/behaviors in terms of difficulty to perform.

Difficulties carrying out such measures were also screened and are displayed in Figure 2. The most difficult to perform COVID-19 preventive measures/behaviors reported by the studied population are displayed in Figure 2, with acceptant individuals referring significantly more difficulties concerning limiting social gatherings (χ^2^ (2, *N* = 300) = 7.666, *p* = 0.022).

One statistically significant model was obtained (χ^2^ (7, *N* = 278) = 124.548, *p* < 0.001) explaining 51.3% (R^2^ Nagelkerke) of attitudes towards vaccination, and correctly classifying 82.7% of the sample (91.8% of accepting individuals and 61.5% of hesitant individuals). Acceptance of COVID-19 vaccine was related to have greater confidence in the COVID vaccine security (OR = 0.599) and effectiveness (OR = 0.683), older age (OR = 0.952), to be a healthcare professional (OR = 0.363), to have vulnerable individuals in charge (OR = 0.330), and sustain the belief that the vaccine will end the pandemic situation (OR = 0.346) or not being sure but give some credence to that belief (OR = 0.414). 

The complete set of findings from the logistic regression analysis for the studied sample is presented in Table 4.

## 4. Discussion

COVID-19 vaccines are considered to be a key to attain herd immunity and flatten the current epidemic curve. However, concern about vaccine hesitancy was growing worldwide, even before the COVID-19 pandemic outbreak, prompting the World Health Organization (WHO) to declare it among the top 10 health threats in 2019 [11]. The success of the COVID-19 vaccination program will rely on the rates of vaccine acceptance among the population, but several determinants influence whether an individual refuses, delays, or accepts some vaccines. This study aimed to describe and characterize rates and profiles of populations accepting of, hesitant about, or resistant to a vaccine for COVID-19 in the context of strict COVID-related gathering and mobility restriction measures.

Results showed that willingness to get vaccinated in our studied sample was medium-to-high (65.44%), especially among the elderly (>60) and within the healthcare professionals collective [11,12]. However, a not insignificant percentage of participants expressed doubts about being vaccinated (27.85%) or were reluctant (6.71%). These rates are especially concerning since vaccines are one of the most evidence-based actions able to stop the disease spread and flatten the curve of the COVID-19 contagion [11]. Therefore, there is an urgent public health need for effective messaging to ensure vaccine uptake in future similar situations.

Compared to those resistant or reluctant to be vaccinated, acceptant individuals suffer more psychological symptoms such as fatigue/tiredness, apathy, stress, depressed mood, anxiety, or irritability, despite differences not reaching statistical significance when analyzing symptom per symptom. Thus, a higher percentage of individuals within this group revealed to have been receiving psychological support due to COVID-19 situation or pre-existing problems during the pandemic. Despite no research studies depicting psychological impact in different profiles of vaccine acceptance, the prevalence of psychological symptoms found in our studied sample is in line with recent meta-analyses already indicating that the most common indicators of psychological impact reported across studies were anxiety (28%–38%) and depression (23%–32%), with individuals with pre-existing conditions showing higher rates [34]. Overall, fear of COVID-19 (FCV-19S) in the studied sample was medium-to-high and, despite statistically significant differences were not found between groups, those suffering from chronic diseases and in charge of vulnerable individuals tend to score higher. In addition, hesitant individuals had slightly superior scores compared to acceptant or resistant ones. Similarly, general anxiety evaluated utilizing the GAD-7 was similar between groups with overall scores around 6 on a 0–21 range, revealing medium-to-low anxiety in the studied sample. However, as previous research already demonstrated, women in charge of vulnerable individuals were significantly more anxious [1,2,5,35]. Besides, in our sample of participants more willing to accept vaccination, a higher proportion of individuals suffering from a chronic disease and grieving due to losing a loved one because of the COVID was found. This late event reaches statistical significance compared to hesitant or resistant individuals where the loss of a loved one due to the infectious disease was anecdotal (2.3% among resistant individuals; 1.3% among hesitant individuals). So far, just a few studies have explored vaccine acceptance rates related to grief due to the COVID-19 pandemic. COVID-19 has led to bereavement in response to the loss of family members, relatives or close friends, and loss of jobs, social proximity, and “normality” in general. For those grieving a loss of any kind, their experience will likely be magnified by both the restrictions due to preventive measures and the intersection of their grief over many losses. Vaccines, as our results have shown, could be perceived as an effective way of protecting oneself—especially in those individuals already considered vulnerable for suffering chronic conditions—but also of helping loved ones, tapping into people’s desire to protect and support their friends and family. In times of uncertainty and strict lockdown, vaccines could be perceived as one promising measure to regain certain normalcy steadily. That seems to be the case for those individuals of our studied sample, referring to their willingness to be vaccinated. They are precisely those who have been more struck by the loss of a loved one and are experiencing more psychological symptoms, with a higher percentage of individuals already on psychological treatment or starting to receive it during the pandemic. However, other research did not find such associations but the contrary. A history of acquaintances having suffered from COVID-19, having been hospitalized, and/or admitted to ICU or even died due to COVID-19 did not have a significant association to vaccine acceptance [36].

When exploring vaccine confidence, a composite of measures on safety, efficacy, effectiveness, and importance of the COVID-19 vaccine as the possible solution to the pandemic was employed. Overall, vaccine confidence was significantly higher among acceptant individuals. In addition, they were more prone to rate better the safety and the effectiveness of the vaccine with means scores above 7.5 points on a 0–10 VAS. In addition, half of the acceptant individuals see COVID vaccines as one direct solution to the COVID-19 situation and getting lives back, drawing on the powerful motivation to return to the activities and people they are missing.

As some previous research has demonstrated, clear and consistent communication by government officials and health institutions is critical in building confidence in vaccine programs among individuals [21,37,38]. These actions necessarily include explaining the development of vaccines understandably, how they work (i.e., the time needed for attaining protection, secondary effects, the importance of population-wide inoculation to achieve herd immunity), and their safety and efficacy rates [39,40,41]. Additionally, previous research about human papillomavirus vaccination has also pointed out that loss-framed messages are more persuasive than gain-framed messages for avoidance-oriented individuals, whereas both frames are equally effective for approach-oriented individuals [21,37,38]. Thus, such frameworks might be interesting to be analyzed in-depth concerning COVID-19 or other infectious diseases vaccine acceptance when designing general population health message campaigns.

Among profiles, and despite not reaching statistically significant differences, compliance with preventive measures/behaviors was clearly higher among individuals more willing to get vaccinated. Moreover, they performed more and more frequently all the measures/behaviors surveyed. In contrast, an evident tendency was observed not to perform or carry out less preventive measures/behaviors within the group of resistant individuals, who interestingly referred to higher self-perceived correctness regarding compliance to such measures.

One of the most significant difficulties regarding preventive measures/behaviors for the acceptant individuals was reduced social gatherings. Reduced contact with once common social connections may initially bring about increased feelings of loneliness and social isolation, which might explain the higher rates of psychological symptoms and the higher willingness to get vaccinated among these acceptant individuals [35].

Concerning resistant individuals, it is clear that a lower-risk perception exists since they refer less COVID-19-related fear, less psychological symptoms, and fewer collateral effects of COVID-19, such as having experienced the loss of a loved one or have been quarantined due to COVID-19. Therefore, understanding risk perceptions about the COVID-19 pandemic among different profiles is critical to foster acceptance of a COVID-19 vaccine and trust in sources of information [42,43]. In this line, a very novel and interesting research has provided preliminary findings showing that awareness of danger combined with an optimistic attitude is essential for compliance with public health regulations [44].

One of the main determinants of the spread of epidemics in human population centers is the degree of compliance with public health regulations. In our studied sample, no differences between profiles were observed. However, males displayed more difficulties limiting their outings to certain hours (that is to say, not totally respecting curfews) and reducing gatherings to small groups and restricting their movements to some geographical regions (regional confinements). Something similar happened with individuals between 18–35 years old who demonstrated more difficulties respecting social distancing and reducing outings. It is worth noting that gender has been established as one of the key social determinants of health [45,46,47,48]. In this sense, risky health behaviors are recognized as expressions of masculinity [46,48,49,50] and therefore, it might explain why they tend to expose more themselves to potential contagious situations compared to females, which remained a predictor of compliance with public health regulations [45,46,47,48]. Besides, those in the youngest age group (18–35) reported more difficulties engaging in social restrictions/distancing behaviors than older respondents. This might be again linked to awareness of danger and self-perceived vulnerability. Our results revealed that this age group was associated with a lower likelihood of intending to get a vaccination than those >60 years old [51]. However, this could also be related to the self-perceived impact that these measures could imply to them. Overall, daily social distancing (close adherence to social distancing guidelines) was associated with decreased psychological well-being/resources, fewer positive health behaviors, and increased reports of stress-related physical illness symptoms [1,35]. These potential negative effects are especially relevant in a developmental stage where the building and prospects regarding the personal and professional life project are in full swing. Despite differing perceptions of risk and impact, adoption and perceived effectiveness were largely similar across age groups with high perceived efficacy and high levels of adoption.

When trying to identify a profile of individuals more prone to accept vaccination, several characteristics were outlined. These were having greater confidence in the COVID-19 vaccine security and effectiveness, older age, being a healthcare professional, having a vulnerable individual in charge, and sustaining the belief that the vaccine will end the pandemic situation or at least consider it a possible solution despite still being skeptical. These results highlight the importance of good awareness and information campaigns for the population with a diminished perception of risk or less responsibility towards the other, either because of their socioeconomic situation or personal and professional situation. Therefore, as shown, the acceptance of the vaccine does not seem a reactive attitude to the fear or anxiety of being infected, but rather to an attitude of social responsibility and awareness that a significant part of the population maintains. Therefore, in similar future situations, it is important to identify and characterize those doubtful or resistant to design strategies that effectively reach this segment of the population, to promote the effectiveness of different universal health prevention campaigns.

### 4.1. Limitations

This study has its limitations owing to the small sample size for exploratory factor analysis and for the time-sensitivity of the outbreak. Besides, with a curfew in place, we adopted a snowballing sampling strategy. However, non-probabilistic sampling techniques make it impossible to determine the sampling error or make solid inferences about populations. Similarly, this technique does not ensure the representativeness of the sample concerning regions and therefore, as it can be observed, a high proportion of respondents (65.3%) were from one region from Spain (Barcelona). Further, the survey provides cross-sectional data at a particular point in time regarding the population’s attitudes towards vaccination. Thus, longitudinal designs with a larger sample size would be desirable, carrying out long-term follow-ups to explore the impact of interventions on mid-to-long-term outcomes. Lastly, the number of respondents who have been quarantined, diagnosed with COVID-19 have been minimal, and there have been no cases admitted to hospital and/or ICU. Interestingly, a high proportion of respondents were healthcare professionals, which can also introduce bias and could limit generalization of results. Therefore, our results could not be generalized to them.

Despite all the above limitations, our study provides very relevant information about populations’ attitudes towards vaccination in a context of strict social gathering and mobility regulations, and at a specific time point where vaccination’s development and implementation were not generalized yet. All this knowledge provides insights toward the barriers and challenges leading to lower vaccine acceptance rates, offering a more comprehensive understanding of how public health officials can effectively tailor health behavior messaging to align with the psychological profiles and dispositions of vaccine-hesitant/resistant individuals, achieving public health campaigns and their objectives in a more efficient manner.

### 4.2. Implications for Future Research

This research project’s findings would help identify, develop, and implement data-driven, evidence-based, and human-centered behavior modification interventions to address COVID-19, or other diseases, and vaccine hesitancy among different population profiles. However, future research must explore how health communication must reach all communities to enhance vaccine literacy to prevent future infections and mortality. Besides, it is necessary to replicate these preliminary findings on larger samples conducting statistical comparisons with similar studies to test and evaluate similarities or differences in the outcomes across different settings and populations. Besides, long-term follow-ups to explore the impact of such interventions on mid-to-long-term outcomes are needed too.

## 5. Conclusions

To our knowledge, this study provides a novel and in-depth understanding of various factors related to COVID-19 vaccine acceptance, resistance, and hesitancy among individuals living with different psychographic and clinical profiles. These results could help identify critical areas that need to be addressed through intervention to enhance vaccine literacy, addressing community-specific misconceptions regarding vaccines, and fostering compliance with health recommendations.

## Figures and Tables

**Table 1:** Participants’ demographics (*N* = 300).

Variables	*n* (%)
**Gender**	
Female	227 (75.7%)
**Country of origin**	
Spain	291 (97%)
Other ^a^	9 (3%)
**Cohabiting**	
Alone	32 (10.7%)
Couple/partner	192 (64%)
Mother	57 (19%)
Father	40 (13.3%)
Children	118 (39.3%)
Siblings	26 (8.7%)
Grandmother	3 (1%)
Grandfather	1 (0.3%)
Mother-in-law	3 (1%)
Father-in-law	1 (0.3%)
Caregiver (formal/informal)	2 (0.7%)
Pet(s)	55 (18.3%)
Flat mates	16 (5.3%)
**Vulnerable individuals at charge**	
Yes	65 (21.8%)
**Age**	
18–35	114 (38%)
36–59	160 (53.3%)
>60	26 (8.7%)
**Occupation**	
Healthcare professionals	131 (43.7%)
**Education**	
Primary school	9 (3%)
Secondary school	21 (7%)
Higher education	52 (17.3%)
University degree	178 (59.3%)
PhD	25 (8.3%)
Other degrees	15 (5%)
**Employment situation**	
Working	243 (81%)
Temporary labor-force adjustment	5 (7.8%)
Dismissal	2 (3.1%)
Unemployed	9 (14.1%)
**Self-perceived socio-economic status**	
Low	52 (17.3%)
Medium	230 (76.7%)
High	18 (6%)

^a^ This includes: UK (*n* = 2), Colombia (*n* = 2), Germany (*n* = 1), Norway (*n* = 1), Malaysia (*n* = 1), Indonesia (*n* = 1), Mexico (*n* = 1).

**Table 2:** Clinical and COVID-19 related data (*N =* 300) ^a^.

Variables	Acceptance *(n =* 195) *n* (%)	Resistant (*n =* 20) *n* (%)	Hesitant (*n =* 83) *n* (%)
Chronic disease (*yes*)	38 (12.8%)	3 (1%)	14 (4.7%)
Loss of a loved one due to COVID-19 (*yes*) *	39 (13.1%)	7 (2.3%)	4 (1.3%)
Receiving psychological support (*yes*)	44 (14.8%)	4 (1.3%)	17 (5.7%)
**Psychological support specification (*yes*)**			
Due to COVID-19	11 (3.7%)	0	1 (0.3%)
Pre-existing problem	17 (5.7%)	4 (1.3%)	9 (3%)
Both	12 (4%)	0	3 (1%)
Other problems	3 (1%)	1 (0.3%)	6 (2%)
**Psychological symptoms experienced related to COVID-19 and the pandemic (*yes*)**			
Anxiety	59 (19.8%)	7 (2.3%)	26 (8.7%)
Stress	69 (23.2%)	8 (2.7%)	26 (8.7%)
Depressed mood	65 (21.8%)	9 (3%)	20 (6.7%)
Panic attacks	3 (1%)	0	2 (0.7%)
Fatigue/tiredness	86 (28.9%)	7 (2.3%)	34 (11.4%)
Apathy	72 (24.2%)	11 (3.7%)	32 (10.7%)
Not willing/interested in talking/contact others	30 (10.1%)	5 (1.7%)	16 (5.4%)
Changes in appetite	32 (10.7%)	5 (1.7%)	13 (4.4%)
Changes in sleep patterns	35 (11.7%)	5 (1.7%)	13 (4.4%)
Irritability	58 (19.5%)	7 (2.3%)	19 (6.4%)
Intense fear	6 (2%)	1 (0.3%)	4 (1.3%)
Intrusive worries	34 (11.4%)	0	14 (4.7%)
General physical malaise/distress	52 (17.4%)	6 (2%)	22 (7.4%)
Other problems/disturbances	6 (2%)	1 (0.3%)	1 (0.3%)
COVID-19 positive diagnosis (*yes*)	32 (10.7%)	0	10 (3.4%)
**Testing (*yes*)**			
Rapid test	3 (1%)	0	1 (0.3%)
CRP	24 (8.1%)	0	9 (3%)
Swab	1 (0.3%)	0	0
Serological testing	16 (5.4%)	0	3 (1%)
Antigen test	8 (2.7%)	1 (0.3%)	1 (0.3%)
Other tests	2 (0.7%)	0	1 (0.3%)
Quarantine due to COVID-19 diagnosis (*yes*)	7 (2.3%)	0	2 (0.7%)
**Quarantine due to COVID-19 possible case (*yes*)**			
1 period (10 days)	26 (57.8%)	0	7 (15.6%)
2 periods (20 days)	5 (11.1%)	0	2 (4.4%)
>2 periods (>20 days)	0	0	1 (2.2%)
**Self-perceived safety (0–10) of COVID-19 vaccines**, M ± SD, range	7.76 ± 1.58, 2–10	2.65 ± 2.56, 0–7	5.42 ± 2.02 0–10
**Self-perceived effectiveness (0–10) of COVID-19 vaccines**, M ± SD, range	7.67 ± 1.53, 2–10	3.25 ± 2.38, 0–8	5.52 ± 2.01, 0–10
**Self-perceived effectiveness of COVID-19 vaccines as other well-established vaccines** (*yes*) *	140 (47%)	5 (1.7%)	37 (12.4%)
**Vaccine as the solution for COVID-19 pandemic** (*yes*) *	108 (36.2%)	3 (1%)	21 (7%)
**Fear of COVID-19 *(FCV-19S*)**, M ± SD, range	16.73 ± 5.43, 7–34	13.80 ± 5.41, 7–24	17 ± 5.17, 7–28
**Generalized anxiety (*GAD-7*)**, M ± SD, range	6.24 ± 5.06, 0–21	5.25 ± 3.58, 0–13	6.12 ± 4.62, 0–21

^a^ Two missing values; * *p* < 0.05 according to the Chi-square test or Mann–Whitney U test.

**Table 3:** COVID-19 preventive measures/behaviors (*N* = 300) ^a^.

Variables	Acceptance (*n* = 195) *n* (%)	Resistant (*n* = 20) *n* (%)	Hesitant (*n* = 83) *n* (%)
Use of FPP2 masks	112 (37.6%)	9 (3%)	47 (15.8%)
Use of other masks	115 (38.6%)	12 (4%)	48 (16.1%)
Use of anti-COVID plastic face shields	13 (4.4%)	1 (0.3%)	5 (1.7%)
Use of anti-COVID transparent shields/physical barriers on the desk/counter	34 (11.4%)	2 (0.7%)	16 (5.4%)
Hand hygiene with hydroalcoholic gel	177 (59.4%)	17 (5.7%)	73 (24.5%)
Surface hygiene with hydroalcoholic gel	99 (33.2%)	10 (3.4%)	44 (14.8%)
Wearing gloves *	34 (11.4%)	4 (1.3%)	3 (1%)
Social distancing (1.5–2 m)	160 (53.7%)	13 (4.4%)	63 (21.1%)
Restriction of departures at certain hours (curfew)	128 (43%)	14 (4.7%)	54 (18.1%)
Limiting distance from home	91 (30.5%)	7 (2.3%)	34 (11.4%)
Limiting social gatherings to small groups	177 (59.4%)	17 (5.7%)	70 (23.5%)
Decreasing the frequency of attending to closed places	162 (54.4%)	15 (5%)	66 (22.1%)
Decreasing the frequency of attending crowded places for leisure	171 (57.4%)	17 (5.7%)	72 (24.2%)
Self-perceived compliance with COVID-19 preventive measures/behaviors (0–5 VAS), M ± SD, range *	4.16 ± 0.62, 3–5	4.20 ± 0.52, 3–5	3.96 ± 0.67, 2–5

^a^ Two missing values; * *p* < 0.05 according to the Chi-square test.

**Table 4:** Binary logistic regression model (*N* = 278).

Hesitant vs. Acceptance ^a^		95% CI for Exp(B)		
	B(SE)	Inferior	OR	Superior
Intercept	8.37 (1.24) ***			
Confidence in the COVID-19 vaccine security	−0.51 (0.14) ***	0.452	0.599	0.794
Age	−0.05 (0.01) ***	0.925	0.952	0.980
Healthcare professional	−1.01 (0.37) **	0.177	0.363	0.744
Confidence in COVID-19 vaccine effectiveness	−0.38 (0.15) *	0.506	0.683	0.923
Vulnerable individuals in charge	−1.11 (0.48) *	0.129	0.330	0.844
Belief that the vaccine will end the pandemic situation				
Yes	−1.06 (0.42) *	0.152	0.346	0.792
Don’t know/No answer	−0.88 (0.44) *	0.175	0.414	0.977

^a^ Reference category Acceptance. R^2^ = 0.361 (Cox & Snell); 0.513 (Nagelkerke). Model (χ^2^ (7, *N =* 278) = 124.548, *p* < 0.001. * *p* < 0.05, ** *p* < 0.01, *** *p* < 0.001.

## Data Availability

The data that support the findings of this study are available from the corresponding author upon reasonable request.

## Supplementary Materials

The following are available online at https://www.mdpi.com/article/10.3390/ijerph182312348/s1, Table S1: The Checklist for Reporting Results of Internet E-Surveys (CHERRIES).
